# Retraction: A novel MADM algorithm for physical education teaching quality evaluation based on 2-tuple linguistic neutrosophic numbers power heronian mean operators

**DOI:** 10.1371/journal.pone.0286613

**Published:** 2023-05-30

**Authors:** The PLOS ONE Editors

After this article was published, similarities were noted between this article and submissions by other research groups which call into question the validity and provenance of the reported results, and the adherence of this article to the PLOS Authorship policy. Further editorial assessment identified concerns regarding the integrity of the peer review process. In light of these issues, the *PLOS ONE* Editors retract this article [1].

FR did not agree with the retraction. MX either did not respond directly or could not be reached.

## References

[1] Rao F, Xiao M (2023) A novel MADM algorithm for physical education teaching quality evaluation based on 2-tuple linguistic neutrosophic numbers power heronian mean operators. PLoS ONE 18(2): e0279534. 10.1371/journal.pone.0279534 doi: 10.1371/journal.pone.0279534 36758011 PMC9910655

